# Variable skeletal phenotypes associated with biallelic variants in *PRKG2*

**DOI:** 10.1136/jmedgenet-2021-108027

**Published:** 2021-11-15

**Authors:** Alistair T Pagnamenta, Francisca Diaz-Gonzalez, Benito Banos-Pinero, Matteo P Ferla, Mehran B Toosi, Alistair D Calder, Ehsan G Karimiani, Mohammad Doosti, Andrew Wainwright, Paul Wordsworth, Kathryn Bailey, Katarina Ejeskär, Tracy Lester, Reza Maroofian, Karen E Heath, Homa Tajsharghi, Deborah Shears, Jenny C Taylor, The Genomics England Research Consortium, John C Ambrose

**Affiliations:** 1 NIHR Biomedical Research Centre, Oxford, Oxfordshire, UK; 2 Wellcome Centre for Human Genetics, Oxford University, Oxford, Oxfordshire, UK; 3 INGEMM, IdiPAZ and Skeletal Dysplasia Multidisciplinary Unit (UMDE, ERN-BOND), Hospital Universitario La Paz, Madrid, Spain; 4 Oxford Genetics Laboratories, Oxford University Hospitals NHS Foundation Trust, Oxford, Oxfordshire, UK; 5 Department of Pediatric Neurology, Ghaem Hospital, Mashhad University of Medical Sciences, Mashhad, Iran; 6 Radiology Department, Great Ormond Street Hospital for Children NHS Foundation Trust, London, UK; 7 Genetics Research Centre, Molecular and Clinical Sciences Institute, St. George’s, University of London, London, UK; 8 Next Generation Genetic Polyclinic, Razavi International Hospital, Mashhad, Iran; 9 Department of Paediatrics, Oxford University Hospitals NHS Foundation Trust, Oxford, UK; 10 School of Health Sciences, Translational Medicine, University of Skövde, Skövde, Sweden; 11 Department of Neuromuscular Disorders, Queen Square Institute of Neurology, UCL, London, UK; 12 CIBERER, ISCIII, Madrid, Spain; 13 Oxford Centre for Genomic Medicine, Oxford University Hospitals NHS Foundation Trust, Oxford, Oxfordshire, UK

**Keywords:** musculoskeletal diseases, genomics, phenotype, frameshift mutation, codon, nonsense

The 100 000 Genomes Project (100KGP) is a UK-wide initiative that has a goal of using whole genome sequencing (WGS) to identify genetic causes of rare inherited diseases and embed the use of this technology within the NHS.^1^ Using data from this resource alongside international gene-matching efforts, four individuals from two independent families were identified harbouring homozygous frameshift or stop-gain variants in *PRKG2*, a recently described skeletal dysplasia gene.^2^ Detailed clinical and radiological assessments helped extend the phenotypic range associated with this autosomal recessive condition while functional studies indicated that both variants had a similar impact on FGF-induced MAPK signalling.

*PRKG2* encodes the cyclic guanosine monophosphate dependent protein kinase II (cGKII), which acts downstream of the natriuretic peptide receptor-B/C- natriuretic peptide (NPR-B/CNP). NPR-B is encoded by *NPR2,* biallelic variants in which are responsible for acromesomelic dysplasia, Maroteaux type (AMDM; MIM 602875). Rodent models further implicate PRKG2 in skeletal development^3 4^ and cGKII deficiency was shown to be the cause of the dwarfism phenotype observed in Angus cattle.^5^ Building on support from pathway analysis and model organisms, a recent study showed that biallelic *PRKG2* variants can result in acromesomelic dysplasia, PRKG2-type (AMDP) in humans,^2^ adding *PRKG2* to a list of >400 genes associated with genetic skeletal disorders.^6^ As only two affected individuals were reported, it is important that the full clinical range of this condition is described.

In this study, we searched for rare biallelic *PRKG2* variants using data from the 100KGP via the LabKey application available within Genomic England’s research environment. Researchers can apply for access online (www.genomicsengland.co.uk/join-a-gecip-domain). Initial filtering employed a 1% population allele frequency threshold based on data from the 1000 Genomes Project as well as in-house frequency information. An additional family was identified via a network of collaborators and variants were classified using ACMG criteria (online supplemental table 1).

10.1136/jmedgenet-2021-108027.supp1 Supplementary data

**Figure 1:**
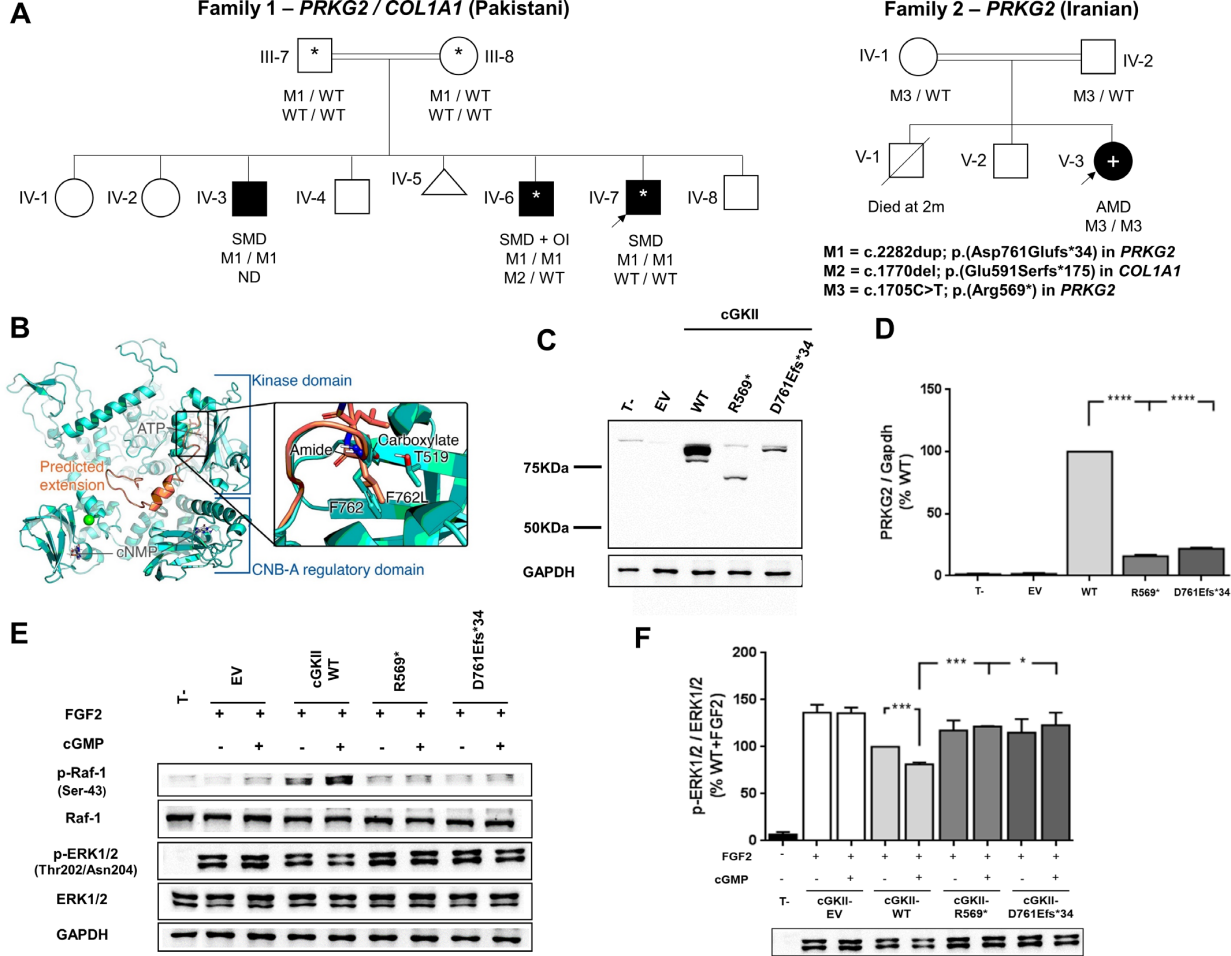
Pedigrees, structural modelling and the effects of the *PRKG2* variants on cGKII protein levels/MAPK pathway regulation. (A) Simplified pedigrees and segregation of variants in *PRKG2* and *COL1A1* in two families with rare skeletal dysplasias. More detailed pedigrees are shown in online supplemental figure 4. AMD, acromesomelic dysplasia (mild); ND, not determined; OI, osteogenesis imperfecta; SMD, spondylometaphyseal dysplasia; WT, wild-type; *, WGS performed as part of 100KGP; +, exome sequencing. The *COL1A1* variant was initially detected by targeted sequencing in 2011 but confirmed to have arisen *de novo* by WGS. (B) Structure of cGKII (wild type: turquoise) with overlay of the mutant, p.Asp761Glufs*34 (salmon) extension and inset of Phe762 residue. The protein kinase domain is regulated by two cyclic nucleotides binding (CNB) domains. The predicted C-terminal extension would fall between CNB-A domain and the protein kinase domain and is likely to interfere with the activation of the latter by the former, were it to be stable, a conclusion not supported by *in silico* predictions. In fact, the extension results in a deleterious amino acid change of a core residue, Phe762, to a leucine (inset). Also visible is the hydrogen bond between the terminal carboxylate and Thr519, whereas the amide bond between Leu762 and Leu763 is forced away in order to best accommodate the subsequent residues. (C) Immunoblotting results for cGKII (upper panel) and GAPDH as an endogenous control (lower panel) of cell lysates extracted from transiently transfected HEK293T cells. Both human cGKII mutants as well as wild-type (WT) proteins were detected at their predicted size: R569*: 65.1 kDa and D761Efs*34: 91.1 kDa (calculated by using the ExPaSy online tool, https://web.expasy.org). (D) Densitometry quantification of cGKII showing that there is an 80% reduction in expression of the two mutants compared with WT. (E) Western blots of phosphorylated Raf-1 and ERK1/2 proteins of the MAPK pathway showed that neither of the mutants were able to phosphorylate c-Raf at Ser43 and therefore downregulate ERK activation compared with WT in response to FGF2 induction in transiently transfected HEKT293 cells. (F) Densitometry quantification of pMAPK 44/42 protein revealed that neither R569* nor D761Efs*34 mutants were able to downregulate FGF2-induced ERK1/2 activation compared with WT, in transiently transfected HEK293 cells in the presence of 8-pCPT-cGMP. Three biological experiments were performed, and significance values are represented as *p<0.05, **p<0.01, ***p<0.001 and ****p>0.0001. EV, empty vector; T−, untransfected cells.

In family 1, WGS and subsequent Sanger sequencing uncovered a homozygous pathogenic *PRKG2* variant, NM_006259.3:c.2282dup (p.Asp761Glufs*34; online supplemental figure 1) in three brothers referred with spondylometaphyseal dysplasia (figure 1A). Interestingly, the middle-affected brother (F1-IV-6) also has type I osteogenesis imperfecta (OI). An early clinical exome sequencing study found that for 4.6% of cases with a molecular diagnosis, more than one gene was contributing to a blended phenotype.^7^ Complex cases such as these are expected to be more common in highly consanguineous families where large regions of homozygosity (ROHs) make up a significant proportion of the genome; however, for F1-IV-6 the secondary diagnosis of OI was due to a *COL1A1* frameshift, which had arisen *de novo*. OI was suspected in this child because of multiple fractures in childhood (arm as an infant, wrist aged 8 and thoracic T6 wedge fracture) combined with blue sclerae. It is certainly possible that the coexistent OI may have had an impact on the severity of the phenotype in this individual, not least because his height was more significantly reduced than for his two brothers and OI (type 1) is a known cause of reduced stature in its own right.

10.1136/jmedgenet-2021-108027.supp2 Supplementary data

In family 2, exome sequencing for a girl with acromesomelic dysplasia revealed a homozygous pathogenic *PRKG2* variant c.1705C>T; p.(Arg569*) (online supplemental figure 2), observed previously in a patient with similar clinical and radiological features.^2^ Comparison of the available genomic data for F2-V-3 and the previously published case was not able to detect a shared haplotype across the *PRKG2* locus. However, exome sequencing has limited resolution to detect small regions of identity by descent and so a founder mutation cannot be ruled out. Given the differing ethnicities and the fact that c.1705C>T lies at a CpG dinucleotide, the recurrence of c.1705C>T being due to separate mutational events seems a more likely scenario.

**Figure 2:**
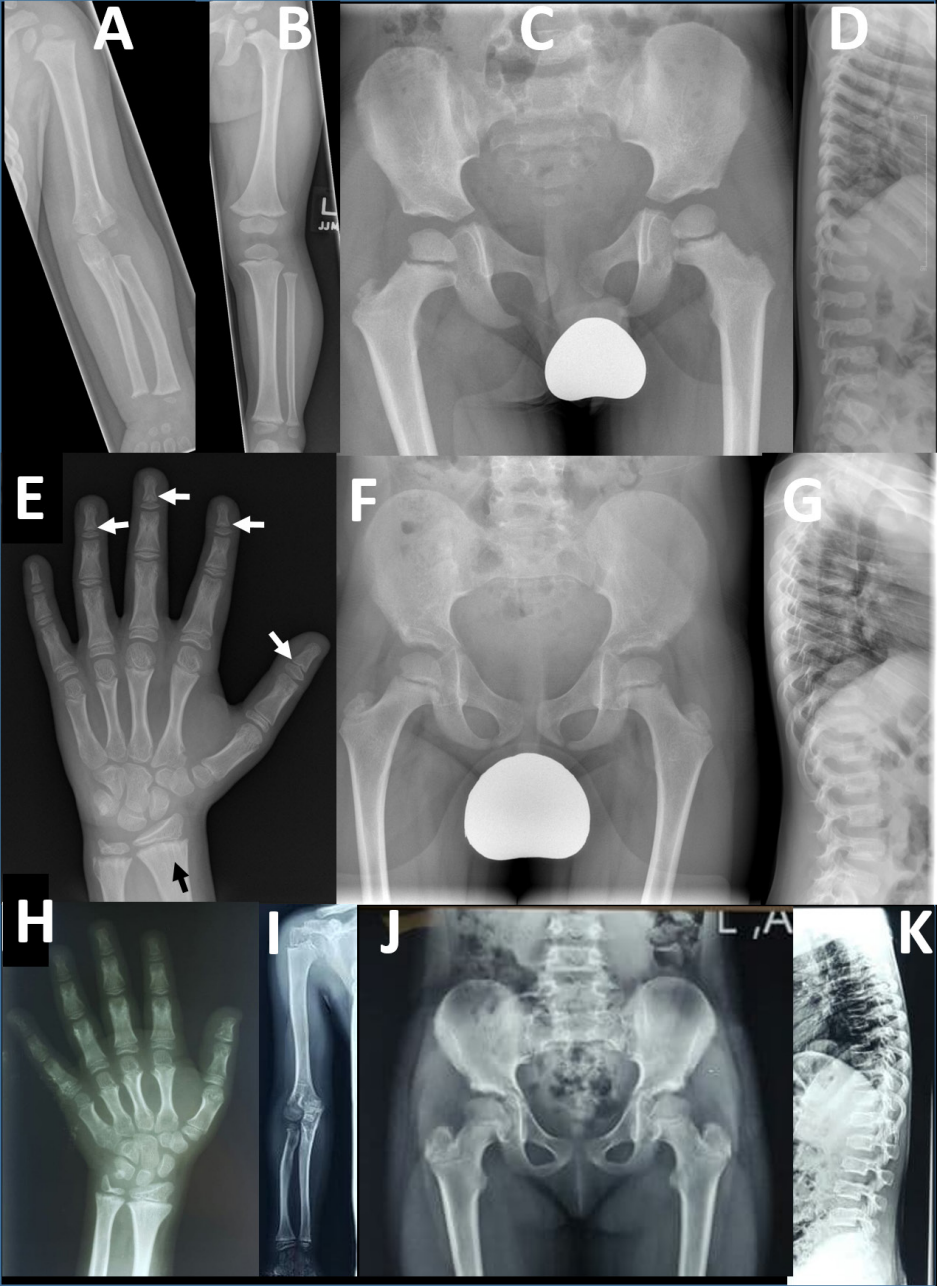
Radiographic findings in two families with *PRKG2* variants: radiographs of left upper limb (A) and lower limb (B) in a 26-month-old boy (F1-IV-7) from family 1. The long bones are stocky in appearance but there is no disproportion within the limbs. (C) Pelvic radiograph at age 4 in same child shows development of long, slender femoral necks. (D) Lateral spinal radiograph at age 4 show generalised mild platyspondyly with small central anterior projections of the vertebral bodies, and hypoplasia of the L2 vertebral body. (E) Left hand radiograph at age 11 in same child shows no brachydactyly; there is mild metaphyseal chondrodysplasia evident in the distal radius and particularly the ulna, with some metaphyseal striations (black arrow); subtle coning of the distal phalangeal metaphyses is evident (white arrows), without associated shortening. Pelvic (F) and lateral spine (G) radiographs in middle affected sibling (F1-IV-6) in family 1 showing similar features of long slender femoral necks and platyspondyly with anterior vertebral body projections. Osteopaenia is also evident; this child also has type 1 osteogenesis imperfecta due to a *de novo* pathogenic variant in *COL1A1*. Additional radiology is available for F1-IV-3 in online supplemental figure 5 which shows similar results to those for F1-IV-7. (H) Left hand radiograph in female child (F2-V-3, aged 10 years) from family 2 showing generalised brachydactyly. (I) Right upper limb radiograph also from F2-V-3 demonstrates mild disproportionate shortening of the radius and ulna relative to the humerus (mesomelic shortening). (J) Pelvic radiograph from F2-V-3 demonstrates mildly elongated femoral necks. (K) Lateral spine radiograph from the same individual demonstrates mild platyspondyly with small anterior vertebral body projections.

Both variants described here are extremely rare; p.(Asp761Glufs*34) is absent from gnomAD (https://gnomad.broadinstitute.org), while p.(Arg569*) is present as a singleton allele. In both families, the disease-causing variants lay within large ROHs (online supplemental table 2). Pathogenic variants are overrepresented in the largest ROHs and it has been proposed that lying in one of the top 10 such regions can be used as evidence supporting pathogenicity.^8^ While the p.(Arg569*) variant has already been demonstrated to affect the downstream MAPK pathway,^2^ the p.(Asp761Glufs*34) in family 1 is likely to be disruptive given the switch of the final Asp-Phe residues for 33 alternative amino acids at the C terminus. *In silico* modelling highlights the structural importance of this region, in particular the final Phe762 residue (figure 1B, supplementary methods; interactive version at https://michelanglo.sgc.ox.ac.uk/r/prkg2).

To functionally confirm the pathogenicity of the newly identified p.(Asp761Glufs*34) variant, we first analysed cGKII expression by western blot analysis. Plasmid construction for p.(Asp761Glufs*34) involved a sequential PCR strategy (supplemental methods), with the previously characterised p.(Arg569*) variant employed as a positive control. For both variants, cGKII was detected at the predicted size (figure 1C), although at dramatically reduced levels (≥80%) compared with the wild type (figure 1D). Next, we evaluated whether the p.Asp761Glufs*34 mutant was able to inhibit FGF2-induced MAPK pathway by analysing its ability to induce phosphorylation of Raf-1 at Ser-43 and ERK1/2, as described previously.^2^ Wild-type cGKII downregulated MAPK signalling by reducing ERK1/2 activation through the upstream phosphorylation of Raf-1 at Ser-43 in a cGMP-dependent manner. However, the p.Asp761Glufs*34 mutant failed to phosphorylate Raf-1 at Ser-43 and thus, reduced FGF2-induced ERK1/2 phosphorylation (figure 1E–F), similar to results for the p.Arg569* variant.^2^

Detailed phenotypic information is provided for both families and compared with the two published cases (online supplemental table 2, figure 3). Radiological findings for F2-V-3 were very similar to those observed for ‘Proband 1’ described previously,^2^ which is unsurprising given that both individuals harbour the same homozygous p.(Arg569*). In contrast, for *PRKG2* family 1 there was a consistent radiological phenotype distinct from previously reported AMDP and AMDM. The three brothers reported here (F1-IV-3, IV-6 and IV-7) had no evidence of acromesomelic shortening, except for mild shortening of toes observed for individual F1-IV-7. The main findings were platyspondyly with anterior vertebral body projections, long slender femoral necks and some metaphyseal irregularity (most evident in the radius and ulna) and striations (figure 2). The metaphyses of the distal phalanges were somewhat cone-shaped in one child, but not pronounced, generalised or associated with shortening, as seen in AMDM.^9^ In summary, family 1 exhibited a skeletal phenotype characterised by spondylometaphyseal dysplasia, rather than acromesomelic dysplasia as expected in AMDP and AMDM.

Interestingly, the *PRKG2* locus has been identified in several genome-wide association studies on height (www.ebi.ac.uk/gwas/genes/PRKG2). Therefore, the description of this now confirmed Mendelian condition constitutes an additional example of rare variants in a gene causing a severe condition, where common variants in the same gene are associated with a related trait.^10^ In summary, analysis of 100KGP data combined with gene-matching efforts identified four affected individuals with biallelic loss of function variants in *PRKG2,* extending the phenotypic range of this condition to include spondylometaphyseal dysplasia. The patients described here were the only individuals harbouring severe biallelic *PRKG2* variants across all rare disease areas within the 100KGP. These data include 295 patients recruited due to an unexplained skeletal dysplasia and therefore our results are consistent with this condition being extremely rare in humans.
