# Supplementary material for: Variable skeletal phenotypes associated with biallelic variants in PRKG2
Source: J Med Genet. 2021 Nov 15;59(10):947–50. doi: 10.1136/jmedgenet-2021-108027 (PMC9554069; doi:10.1136/jmedgenet-2021-108027)
Supplement: Supplementary data [file jmedgenet-2021-108027supp001.pdf]

Table S2: Clinical details and variant information for four individuals from two independent families with biallelic variants in *PRKG2* . Results are compared to two cases reported by Díaz-González *et al* 2020.
Sequencing methods in Family 2 are as described by Pagnamenta *et al* 2021. NA, not available; ROH, region of homozygosity. Variant annotation is based on NM_006259.3. SDs for adults heights were calculated using https://tall.life/height-percentile-calculator-age-country. For Family 1 we used the mean adult height obtained from the Royal College of Paediatrics and Child Health's standard growth chart.

	**Families reported here**				**Díaz-González *et al* 2020**	
**Family/individual ID**	**Family 1**			**Family 2**	**Proband 1**	**Proband 2**
**Consanguinity (parental relationship)**	Yes (double 1st cousins)			Yes (double 2nd cousins)	Yes (3rd cousins)	Yes (3rd cousins)
**Ethnicity**	Pakistani			Iranian	Moroccan	Indian
**Parents' stature**	Father 177 cm (0 SD);<br>Mother 160 cm (-0.60 SD)			Father 155 cm (-2.10 SD);<br>Mother 171 cm (1.90 SD)	Father 165 cm (-1.75 SD);<br>Mother 164 cm (-0.01 SD)	Father 158 cm (-2.59 SD);<br>Mother 147 cm (-2.50 SD)
**Individual**	IV-3	IV-6	IV-7	V-3	II-2	II-2
**Gender (M/F)**	M	M	M	F	F	F
**Age at first referral**	13 years	8 months	2 years	3 years	NA	4 years
**Referral reason**	Diagnosis due to sibling	Poor weight gain, unusual limb proportions, hypotonia, large head	Short stature	Short stature/dysmorphism	Short stature/suspected skeletal dysplasia	Short stature/suspected skeletal dysplasia
**Homozygous variant**	c.2282dupA; p.(Asp761Glufs*34)			c.1705C>T; p.(Arg569*)	c.1705C>T; p.(Arg569*)	c.491dupA; p.(Asn164Lysfs*2)
**Allele Frequency (gnomAD 2.1.1)**	Absent			1/250704	1/250704	Absent
**ROH region GRCh38 (size, rank)**	NA	chr4:25,732,624-85,677,887 (60.0Mb, #1)	chr4:53,687,014-85,677,887 (32.0Mb, #3)	chr4: 30,724,365-89,247,731 (58.5Mb)	NA	NA
**Hypotonia**	NA	Yes	N/A	No	-	-
**Birth weight**	2700 g	N/A	N/A	3350 g	NA	NA
**Birth length**	N/A	N/A	N/A	50 cm	NA (−1.93 SDS at 3 weeks)	NA
**Birth OFC**	N/A	N/A	N/A	34 cm	NA (−0.42 SDS at 3 weeks)	NA
**Developmental milestones**	Normal	Normal	Normal	Normal	Normal	Normal
**Growth / feeding**	No concerns until 4y	Poor feeder	NA	Normal	NA	NA
**Age at last assessment (years)**	26	22	15	10	12	11
**Height (Percentile/SD)**	158.5 cm (-2.5 SD)	140.8 cm (-4.9 SD)	143.8 cm (-3.11 SD)	121 cm (-2.5 SD)	-4.01 SD	−5.06 SD
**Sitting height (cm)**	85	76	77	70	NA	NA
**Leg length**	NA	-2 to -3 SD	NA	53	NA	NA
**Skeletal proportions**	Mild rhizomelia of upper and lower limbs	Moderate rhizomelia of upper and lower limbs	Mild rhizomelia of upper and lower limbs	Rhizomelic shortening of the upper and lower extremities.	Mild mesomelic shortening of the limbs (lower limbs, radius and ulna); sitting height to height ragio was 0.579 (1.21 SDs)	No (upper to lower segment ratio 1.41 aged 11y)
**OFC (centile)**	50-75th	50-75th	50-75th	56cm	-	-
**Kyphosis**	Yes, mild, thoracic region	Yes, mild, mid-thoracic	-	No	-	-
**Digital anomalies**	Normal except for broad thumbs and short 4-5th metacarpels	Normal except for broad thumbs	-	Short, broad fingers	Short, stubby fingers	Short stubby fingers
**Acromelia**	No	No	No	No	-	-
**Feet**	Flat feet	NA	Mild shortening of toes (Figure S3A bottom)	Short broad toes	-	Sandal gap
**Facial appearance**	Normal	Triangular face, when younger	Normal	Broad nasal bridge, thick eyebrows, synophrys, prominent chin	Normal	Triangular face, broad nasal bridge, pointed chin, synophrys, hypertelorism, low set ears.
**Cranial findings**	NA	Multiple Wormian bones	NA	Normal	Normal	Normal
**Palate**	Normal	Normal	Normal	Normal	-	-
**Platyspondyly**	Yes	Yes	Yes (mild)	Yes	Yes (mild flattening of the thoracic vertebral bodies)	Moderate platyspondyly with anterior beaking of vertebral borders of dorsolumbar spine
**Metaphyseal changes**	Yes (widespread)	Yes	Yes (broadened and irregular)	Yes	-	Long bones showed relatively large epiphyses and widening, with some irregularity of the metaphyses. Metaphyseal irregularity of metacarpals/metatarsals
**Other radiological findings**	-	Slender bones with thin cortices, mild bowing of femur, small irregular femoral heads	-	Short metacarp and metatarsal + disostosis peripheral	Mild thoracic scoliosis, lumbar hyperlordosis, short pedicles of the lumbar spine, very mild flaring of the metaphyses and mild genu valgum. Growth plate of knee was prematurely fused, radius and ulna mildly bowed; short broad phalanges and metacarpals (especially 3-5th), all prematurely fused	Prominent deltoid tuberosities of the humeri, short and broad phalanges, ilia short with flaring of the iliac wings, vertebral alterations less prominent and restricted to the thoracic region with mild shortening of the pedicles of her lumbar spine. Pelvic radiograph showed minor irregularity of the acetabula
**Bone age**	Delayed	NA	NA	No delay (9 years at calendar age of 9)	Advanced (+2.6 SD)	Within normal limits
**Bone density**	Normal	NA	Normal	NA	NA	NA
**Suggested diagnoses prior to genetic diagnosis**	Spondylometaphyseal dysplasia (SMD)-type not classified	Spondylometaphyseal dysplasia (SMD)-type not classified and Osteogenesis Imperfecta type 1	Spondylometaphyseal dysplasia (SMD)-type not classified	Pseudoachondroplasia	Similar to AMDM but with milder radiological phenotype, no cone-shaped epiphyses in the hands and relatively mild mesomelia	-
**Other information**	-	Multiple fractures (arm as infant, wrist aged 8y, wedge T6 vertebra), blue sclerae	-	Constipation, umbilical hernia in the past (now normal), hypertrichosis	-	Hirsutism, prominent costochondral cartilages, sternal prominence, widening of wrists, genu varum
**Cardiovascular**	-	-	-	Normal	Complete atrioventricular block (Mobitz IIa)	-
**Treatment**	Good response seen in 6m GH trial aged 14 and a half years. During this time his annual growth velocity increased significantly (3.4 to 7.6 cm/yr).	Pamidronate	Successful trial of Growth Hormone (treatment ongoing) and showing good response (currently 4.6 cm/year).	Somatropin injections (3 months)	Femoral and humeral limb lengthening	-
**Sequencing method**	Sanger sequencing	150 bp paired end sequencing on HiSeqX as part of 100K Genomes Project, Truseq PCR-Free High Throughput library. Sequenced with parents as quod.		Exome sequencing as described previously (PMID: 33559681)	Trio exome sequencing using Sure Select Human All exon V6 targeted capture (Agilent Technologies), paired-end 150 bp sequencing on NovaSeq6000	Nextera Expanded exome kit (Illumina) and paired-end 100 bp sequencing on a HiSeq2000
**Genetic testing prior to WGS/exome**	-	*COL1A1/COL1A2* testing detected *COL1A1:* NM_000088.4:c.1770del; p.(Glu591Serfs*175) *de novo.* Normal karyotype.	Array CGH (60K), *SHOX* sequencing and MLPA all normal	-	Custom-designed NGS skeletal dysplasia panel (327 genes)	-
