# Supplementary material for: Variable skeletal phenotypes associated with biallelic variants in PRKG2
Source: J Med Genet. 2021 Nov 15;59(10):947–50. doi: 10.1136/jmedgenet-2021-108027 (PMC9554069; doi:10.1136/jmedgenet-2021-108027)
Supplement: Supplementary data [file jmedgenet-2021-108027supp002.pdf]

SUPPLEMENTARY INFORMATION

Variable skeletal phenotypes associated with biallelic variants in *PRKG2*

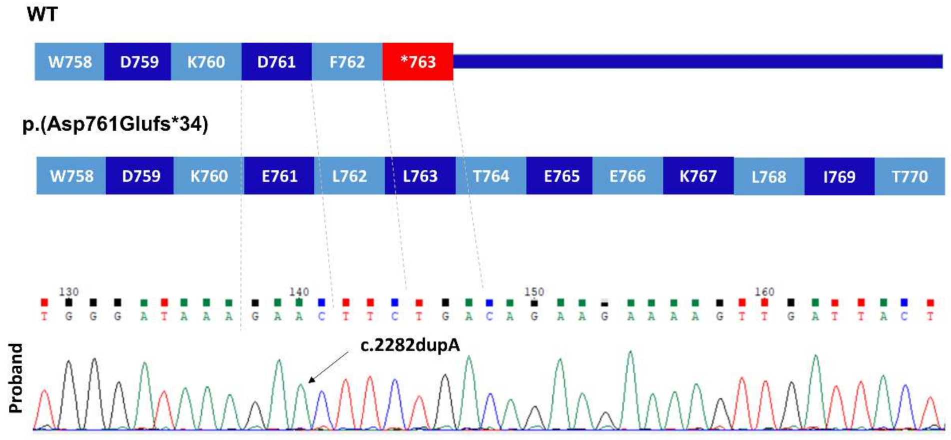

Figure S1: Sanger sequencing electropherogram validating the *PRKG2* variant in Family 1 NM_006259.3: c.2282dupA; p.(Asp761Glufs*34) which lies close to the wild-type C-terminus. Heterozygosity in parental samples was already confirmed by WGS.

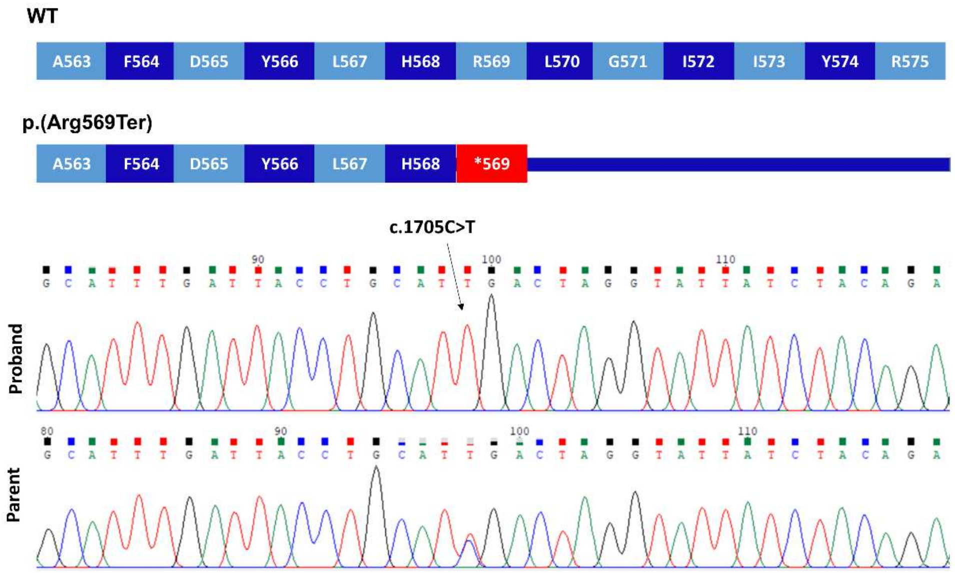

Figure S2: Sanger sequencing electropherogram showing the *PRKG2* variant in Family 2 NM_006259.3: c.1705C>T; p.(Arg569*) to be homozygous in the proband and heterozygous in a parental sample.

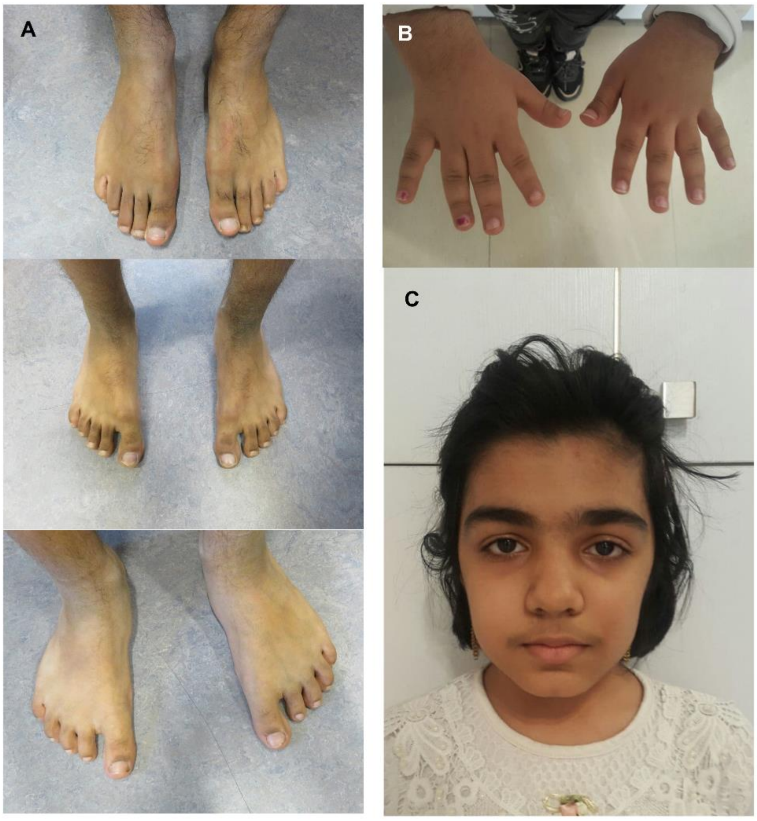

Figure S3: Photos showing clinical features of note. A) Photo showing of the toes in Family 1 which were unremarkable for F1-IV-3 (top) and F1-IV-6 (middle), but with mild shortening seen for F1-IV-7 (bottom). B) Photo showing short/broad fingers in individual F2-V-3 (aged 10 years). C) Photo showing subtle dysmorphic features in F2-V-3, with thick eyebrows, synophrys and a broad nasal bridge.

Pagnamenta AT, *et al. J Med Genet* 2022; 59:947–950. doi: 10.1136/jmedgenet-2021-108027

Recessive skeletal dysplasia associated with *PRKG2*

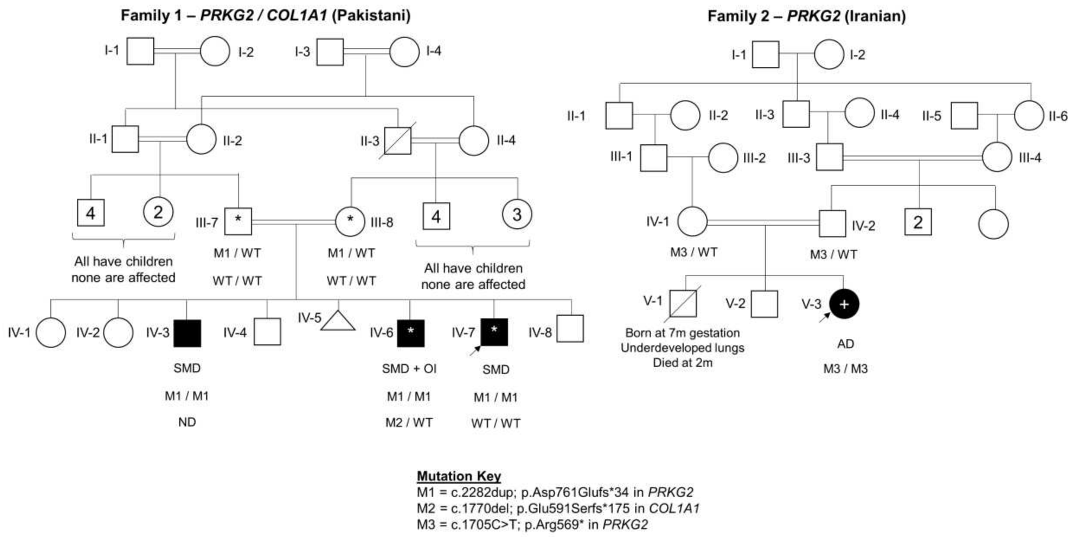

Figure S4: Full pedigrees and segregation of variants in *PRKG2* and *COL1A1* for 2 families with rare skeletal dysplasias. Pedigrees are shown in same order as the simplified versions in Figure 1A. SMD, Spondylometaphyseal dysplasia; OI, Osteogenesis imperfecta; AD, Acromesomelic dysplasia (mild); WT, wild-type; ND = not determined; *, genome sequencing performed as part of 100KGP, +, exome sequencing.

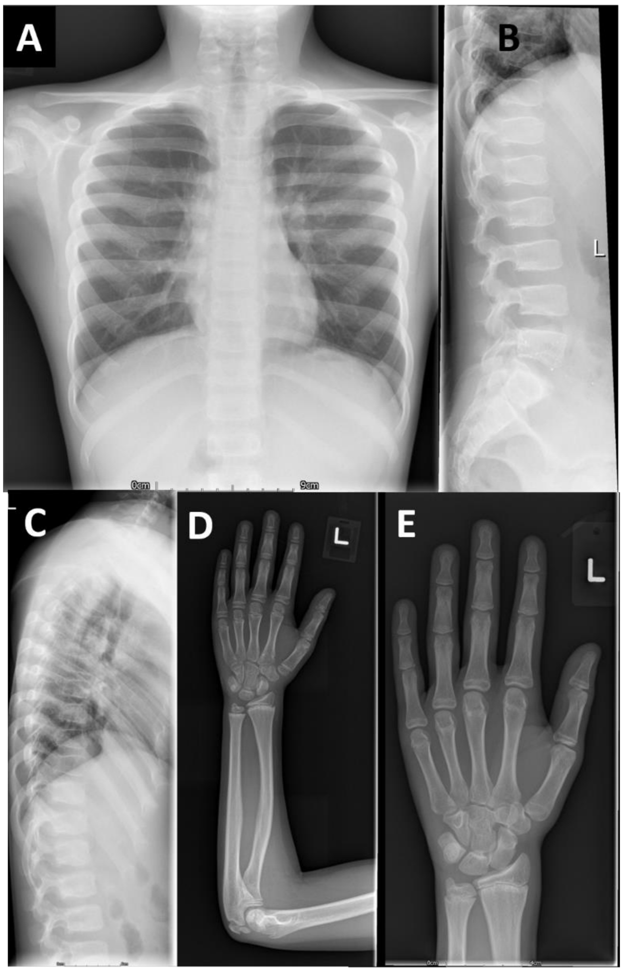

Figure S5: Additional radiological images for individual F1-IV-3. A) Chest radiograph aged 13; normal appearances. Lateral lumbar spine (B) radiograph and lateral thoracic spine radiograph aged 13. There is mild generalised platyspondyly with more pronounced height reduction in the dorsal aspects of the vertebral bodies. D) Hand and forearm aged 13. E) Hand and wrist aged 18; there is no brachydactyly.

Supplementary Methods

In silico modelling of PRKG2 frameshift variant

This composite model of the cGMP-dependent protein kinase 2 was obtained by combining several parts. The kinase domain was predicted by I-Tasser[1], while the cGMP binding domains were the crystal structures PDB:5C6C and PDB:5BV6.[2] The *in silico* calculations were done with Pyrosetta.[3] Further details and the full code is available at https://github.com/matteoferla/PRKG2_analysis. The interactive version of the model is provided using the MichelaNGLo tool.[4]

Plasmid construction

The empty Myc-DDK-tagged expression pCMV6-Entry plasmid as well as the human *PRKG2* (NM_006259.2; pCMV6-PRKG2-WT) wild type plasmids were obtained from Origene Technologies (Rockville, MD). The PRKG2 p.R569* mutant was generated as previously reported.[5] The p.D761Efs*34 mutant was generated by two sequential PCRs in order to add the extra nucleotide sequence to the C-terminus of the wild type sequence. The PCR-1 primers were 5' CCGCCGCGATCGCCATGGGAAATGGTTCAGTGAAACCTAAACATTCTA-AGCACCCAGATGGACAC3' (forward) and 5' CTTCTGTAGAGTACAGGCAGTAATCAACTTTT-CTTCTGTCAGAAGTTCTTTATCCCAGCCTGATAGC 3´ (reverse). The product of PCR-1 was then submitted to a second PCR (PCR-2) to add the remaining nucleotides to complete the mutated sequence. The same forward primer was used in PCR-2 whilst 5' GTACGCGTTAATACTCTGAAAAGAAAATAATGTGTTGGATTATTG-ATCCTTGAGGTCCTCTTCTGTAGAGTACA 3´ was employed as the reverse primer. The PCR product was then cloned into a pCR2.1 vector by TA cloning method following manufacturer´s instructions (Life Technologies, Carlsbad, CA). Next, the cloned fragment was subcloned into pCMV6 vector using the pCMV6-PRKG2-WT construct digested with SgfI and MluI enzymes (Thermo Fisher Scientific) resulting in the pCMV6-PRKG2-D761Efs*34 mutant plasmid. The construct was verified by Sanger sequencing.

MAPK pathway analysis

Cell culture, transient transfections, Western blots and densitometry quantification were performed as previously described.[5]

Table S1: Variant interpretation using ACMG guidelines.[6]

**Family ID**	Family 1	Family 2
**Variant**	NM_006259.3:c.2282dup p.(Asp761Glufs*34)	NM_006259.3:c.1705C>T p.(Arg569*)
**ACMG evidence codes**	PVS1 (applied with caution as near 3' end but N.B. 33 additional residues and WB data in Fig 1C,D supports use), PS3 (MAPK signalling affected, Fig 1E,F), PM2 (absent in gnomAD), PP1 (cosegregation, Fig 1A), PP3 (structural modelling, Fig 1B)	PVS1, PS3 (Diaz-Gonzalez *et al*[5] and now replicated), PM2 (singleton allele in gnomAD 2.1.1), PP3 (*in silico* e.g. CADD=37)
**ACMG classification**	Pathogenic	Pathogenic

Table S2: Clinical details and variant information for four individuals from two independent families with biallelic variants in *PRKG2*. Results are compared to two cases reported by Díaz-González et al 2020.[5] Sequencing methods in Family 2 are as described by Pagnamenta *et al* 2021.[7] NA, not available; ROH, region of homozygosity. Variant annotation is based on NM_006259.3. SDs for adults heights were calculated using https://tall.life/height-percentile-calculator-age-country. For Family 1 we used the mean adult height obtained from the Royal College of Paediatrics and Child Health's standard growth chart

N.B. This table is provided as a separate xlsx file.

References

1 Yang, J. & Zhang, Y. I-TASSER server: new development for protein structure and function predictions. *Nucleic Acids Res* **43**, W174-181, doi:10.1093/nar/gkv342 (2015).

2 Campbell, J. C. *et al.* Structural Basis of Cyclic Nucleotide Selectivity in cGMP-dependent Protein Kinase II. *J Biol Chem* **291**, 5623-5633, doi:10.1074/jbc.M115.691303 (2016).

3 Chaudhury, S., Lyskov, S. & Gray, J. J. PyRosetta: a script-based interface for implementing molecular modeling algorithms using Rosetta. *Bioinformatics* **26**, 689-691, doi:10.1093/bioinformatics/btq007 (2010).

4 Ferla, M. P., Pagnamenta, A. T., Damerell, D., Taylor, J. C. & Marsden, B. D. MichelaNglo: sculpting protein views on web pages without coding. *Bioinformatics* **36**, 3268-3270, doi:10.1093/bioinformatics/btaa104 (2020).

5 Diaz-Gonzalez, F. *et al.* Biallelic cGMP-dependent type II protein kinase gene (PRKG2) variants cause a novel acromesomelic dysplasia. *J Med Genet*, doi:10.1136/jmedgenet-2020-107177 (2020).

6 Richards, S. *et al.* Standards and guidelines for the interpretation of sequence variants: a joint consensus recommendation of the American College of Medical Genetics and Genomics and the Association for Molecular Pathology. *Genet Med* **17**, 405-424, doi:10.1038/gim.2015.30 (2015).

7 Pagnamenta, A. T. *et al.* An ancestral 10-bp repeat expansion in VWA1 causes recessive hereditary motor neuropathy. *Brain* **144**, 584-600, doi:10.1093/brain/awaa420 (2021).
